# Neutrophil trogocytosis during their trans-endothelial migration: role of extracellular CIRP

**DOI:** 10.1186/s10020-022-00515-3

**Published:** 2022-08-08

**Authors:** Satoshi Takizawa, Yongchan Lee, Asha Jacob, Monowar Aziz, Ping Wang

**Affiliations:** 1 Center for Immunology and Inflammation, The Feinstein Institutes for Medical Research, 350 Community Drive, Manhasset, NY 11030 USA; 2 Departments of Molecular Medicine and Surgery, Zucker School of Medicine at Hofstra/Northwell, Manhasset, NY USA

**Keywords:** eCIRP, Trogocytosis, Neutrophils, Trans-migration, Acute lung injury, Inflammation

## Abstract

**Background:**

Neutrophils are the most abundant innate immune cells in the circulating blood, and they act as the first responder against bacterial and fungal infection. However, accumulation of activated neutrophils can cause severe inflammation and tissue damage. Recently, neutrophil trogocytosis or membrane transfer with neighboring cells was reported to modulate immune responses. Extracellular cold-inducible RNA binding protein (eCIRP) is a newly identified damage-associated molecular pattern (DAMP). eCIRP can activate neutrophils to be more pro-inflammatory. This study aimed to identify the role of eCIRP in neutrophil trogocytosis during their trans-endothelial migration.

**Methods:**

A trans-endothelial migration (TEM) assay using bone marrow neutrophils and mouse primary lung vascular endothelial cells was conducted using transwell chambers and neutrophil trogocytosis was assessed in vitro. In an in vivo mouse model of acute lung injury, neutrophil trogocytosis was assessed from bronchoalveolar lavage fluid.

**Results:**

In TEM assay, the trogocytosis of neutrophils occurred during trans-endothelial migration and eCIRP significantly increased the percentage of these neutrophils. The trogocytosed neutrophils acquired the endothelial membrane containing junctional adhesion molecule-C (JAM-C) and VE-cadherin, and these membrane patches were polarized by Mac-1 binding. Furthermore, eCIRP-induced JAM-C positive trogocytosed neutrophils are more pro-inflammatory than the JAM-C negative counterpart. JAM-C positive trogocytosed neutrophils were also observed in the bronchoalveolar lavage fluid of a mouse model of acute lung injury.

**Conclusion:**

These data suggest that during the paracellular trans-endothelial migration of neutrophils in response to inflammation, eCIRP induces trogocytosis of neutrophils, and the trogocytosed neutrophils exhibit an exaggerated pro-inflammatory phenotype promoting acute lung injury.

**Supplementary Information:**

The online version contains supplementary material available at 10.1186/s10020-022-00515-3.

## Introduction

Neutrophils are innate immune cells that act as a front-line responder to cellular injury during infection and inflammation (Amulic et al. 2012). They are recruited to the site of injury and have a key role in response to bacterial or fungal pathogens. However, excessive accumulation of activated neutrophils can cause serious tissue injury and vascular damage (Kolaczkowska and Kubes 2013). Neutrophils are multi-potential cells exhibiting varied biological functions including phagocytosis, production of cytokines/chemokines and the release of neutrophil extracellular traps (NETs). A newly-reported function of neutrophils is trogocytosis or membrane transfer with neighboring cells, to modulate innate and adaptive immune responses (Kruger et al. 2015; Tsai 2021). Trogocytosis has been observed in many organisms including protozoa, nematode, and many different cell types in higher organisms (Uribe-Querol and Rosales 2021). There are different modes of trogocytosis observed including cell killing or cell-to-cell communication (Miyake and Karasuyama 2021). Neutrophils have shown to trogocytose MHC-II antigens from allogeneic B cells and then present MHC-II to allogeneic T cells to enhance proliferation and cytokine production of T cells (Whale et al. 2006). Autologous CD4^+^ T cells trogocytose lactoferrins expressed on neutrophils to skew the T cells towards a T-helper 2 immune phenotype (Wu et al. 2016). These and other studies show that the membrane transfer between neutrophils and other immune cells can modulate immune responses. When neutrophils migrate to the site of injury during inflammation, they need to crosstalk with non-immune cell types such as endothelial cells. In order for the recruiting neutrophils to cross endothelial structure in venules, multiple steps are required, including trans-endothelial migration (TEM) and the involvement of many adhesion molecules (Ley et al. 2007; Nourshargh and Alon 2014; Muller 2011). However, during paracellular trans-endothelial migration, it is not known whether neutrophils trogocytose from the endothelial cells and if so, whether there are any molecular factors which promote neutrophil trogocytosis.

Cold-inducible RNA-binding protein (CIRP) was originally discovered as a RNA binding chaperone that stabilizes RNA molecules in response to cold stress and regulates many cellular processes (Aziz et al. 2019; Nishiyama et al. 1997). Extracellular CIRP (eCIRP) was recently identified as a damage-associated molecular pattern (DAMP) promoting inflammation and organ injury (Qiang et al. 2013). In response to hypoxia, CIRP translocates from the nucleus to the cytosol, then released out of the cells. Since the initial discovery of eCIRP as a DAMP in macrophages, the proinflammatory role of eCIRP has been verified in different cell types including neutrophils (Aziz et al. 2019). Recent studies revealed that eCIRP increased ICAM-1^+^ neutrophils phenotype in bone marrow neutrophils and those ICAM-1^+^ neutrophils exhibited NETosis (Ode et al. 2018). The frequencies of inducible nitric oxide synthase (iNOS) producing NETs were higher in eCIRP treated ICAM-1^+^ neutrophils than ICAM-1^−^ neutrophils (Ode et al. 2018). In another in vivo study, eCIRP treatment increased the expression of CXCR4, ICAM-1, iNOS and NETs in CD11b^hi^ low-density neutrophils in the blood (Takizawa et al. 2021). Furthermore, both sepsis and eCIRP treatment in vivo induced neutrophil reverse TEM by increasing neutrophil elastase and decreasing endothelial junctional-adhesion molecule-C (JAM-C) (Jin et al. 2019). These observations clearly demonstrate that eCIRP induces pro-inflammatory phenotype in neutrophils.

In the current study, we hypothesize that neutrophils undergo trogocytosis from endothelial cells during paracellular TEM and eCIRP not only increases such occurrence but render the trogocytosed neutrophil pro-inflammatory during cellular injury and inflammation. To test this, eCIRP’s effect on neutrophil trogocytosis was evaluated in vitro with a TEM assay and in vivo using an acute lung injury model.

## Materials and Methods

### Animals

C57BL/6 male mice (8–12 weeks old) were purchased from Charles River Laboratories (Wilmington, MA). Mice were housed in temperature-controlled environment with 12-h light/dark cycle and fed a standard laboratory rodent chow and drinking water ad libitum. All animal experiments were performed following the National Institutes of Health guidelines for the care and use of laboratory animals. This study was approved by the Institutional Animal Care and Use Committee of the Feinstein Institutes for Medical Research.

### Antibodies

Purified anti-mouse CD16/32 antibody (Cat. no: 101312, Lot B298973), Alexa Fluor 488 conjugated anti-mouse Ly6G Ab (Cat. no: 127626, Lot B287100), PE conjugated anti-mouse Ly6G Ab (Cat. no: 127608, Lot B290416), Alexa Fluor 488 conjugated Rat IgG2a, κ Isotype control antibody (Cat. no: 400525, Lot B228070), PE conjugated Rat IgG2b, κ Isotype control antibody (Cat. no: 400608, Lot B170512), PE conjugated Mouse IgG1, κ Isotype control antibody (Cat. no: 400139, Lot B244868), PerCP/Cyanine5.5 Rat IgG2a, κ Isotype control antibody (Cat. no: 400531, Lot B268003) were purchased from Biolegend (San Diego, CA). PE conjugated anti-mouse ICAM-1(G-5) Ab (Cat. no: sc-8439; Santa Cruz Biotechnology, Santa Cruz, CA). Anti-VE-cadherin (F-8) (Cat. no: sc-9989 Lot J0217) (Santa Cruz Biotechnology, Santa Cruz, CA). Anti-VE-cadherin (D87F2) (Cat. no: 2500) (Cell Signaling Technology, Danvers, MA). PE Rat anti-mouse CD11b (Cat. no: 557397, Lot 7198506, BD Biosciences, San Jose, CA). APC-conjugated anti-mouse JAM-C(CD323) Rat IgG2b (Cat. no: FAB7050A, Lot ACHX0220011, R&D Systems, Minneapolis, MN). Alexa Fluor 568 goat anti-mouse IgG(H + L) (Cat. no: A11031, Lot 2216598, Invitrogen, Carlsbad, CA).

### Neutrophil isolation

Mouse femur and tibia were used to harvest bone marrow. Briefly, the femur and tibia were isolated, and the bone marrow was flushed out with chilled RPMI media (Thermo Fisher Scientific, Waltham, MA) using a 25 G needle with a syringe. The cell suspension was then filtered with 100 µm mesh cloth and subjected to neutrophil enrichment. Neutrophil enrichment was done with EasySep™ Mouse Neutrophil Enrichment Kit (STEMCELL Technologies, Vancouver, BC).

### Cell culture

Mouse Primary Lung Micro-Vascular Endothelial Cells (MLVEC) were obtained from Cell Biologics (Cat. no. C57-6011, Chicago, IL) and maintained in complete mouse endothelial cell medium (Cell Biologics, Chicago, IL). Cells with passage numbers 5 and 6 were used for all experiments. All cells were maintained at 37 °C, 5% CO_2_ in humidified tissue culture incubator.

### Trans-endothelial migration (TEM) assay

TEM was performed with a transwell chamber assay. Falcon® Permeable Support for 24-well Plate with 3.0 µm Transparent PET Membrane (Corning, Glendale, AZ) was used as the cell culture chamber insert. Insert membrane was coated with gelatin-based coating solution (Cell Biologics, Chicago, IL) for 2 h in the cell culture incubator (37 ℃, 5% CO_2_) and washed once with phosphate buffered saline (PBS) prior to seeding the endothelial cells. 50,000 endothelial cells were seeded in each insert chamber containing 500 µL of endothelial cell culture medium. Then, the chamber insert was placed in a 24-well plate containing 500 μL of endothelial cell culture medium in each well. The endothelial cell culture was maintained in the incubator (37 ℃, 5% CO_2_) for 48 h so that the endothelial cells form a monolayer on the insert chamber membrane. The medium was then removed carefully from the insert. The endothelial cells on the insert chamber were stained with PKH67 using Green Fluorescent Cell Linker Kit for General Cell Membrane Labeling (Sigma, St. Louis, MO) or MemBrite™ 488 dye (Membrite Fix 488/515 Cell Surface Staining Kit, Biotium, Fremont, CA) according to the manufacturer’s protocols. After staining, the insert chamber was immersed in fresh PBS twice to wash away the remaining dye and replenished with 500 µL RPMI medium. Insert chambers were transferred to a separate 24-well plate containing 500 μL of RPMI with 1 nM fMLP (N-formyl-Met-Leu-Phe; Sigma, St. Louis, MO). The purified neutrophils (1 million cells/insert) pre-stained with CellTrace Violet or Yellow (Cell Proliferation Kit, Invitrogen, Carlsbad, CA) were then added to the insert chamber with either PBS or recombinant murine CIRP (rmCIRP, 0.5 µg/mL). rmCIRP was expressed and purified in-house (Qiang et al. 2013). After 4 h incubation in the cell culture incubator, the transmigrated neutrophils in the bottom well were harvested by centrifugation (300×*g* for 10 min) and immediately mounted on the glass slide for either microscopic analysis or immunofluorescence staining. Alternatively, confocal live images were collected during TEM with eCIRP for 2 h and the 3-D volume view image was reconstructed from the z-stack of the confocal microscopic images.

### Confocal microscopy

High resolution images of neutrophil trogocytosis were obtained by Axio Observer.Z1/7 equipped with Zeiss LSM900 confocal microscopy system. The z-stack images of cells were acquired with Plan-Apochromat 63 ×/1.40 Oil DIC M27 objective lens. The imaging of neutrophil trogocytosis were quantified by Zeiss Axio Observer equipped with LSM880 confocal microscopy system. The z-stack images were obtained using 63 ×/1.40 Oil DIC M27 objective lens. SR-4Y fast acquisition mode of Airyscan 2 and 4 × averaging were used. The images obtained by confocal microscope were merged and combined by Fiji ImageJ for quantification (Schindelin et al. 2012).

### Immunofluorescence staining

For the immunostaining of neutrophils collected from TEM assay, neutrophils were spun down at 300×*g* at 4 ℃ for 10 min and the supernatant was discarded. The Fc receptors of neutrophils were first blocked with the buffer containing purified anti-mouse CD16/32 antibody (Biolegend, San Diego, CA). Neutrophils were then stained with appropriate primary antibodies and secondary antibodies for 1 h each at room temperature. After staining, the neutrophils were resuspended with VECTASHIELD Plus Antifade mounting medium with DAPI (Vector Laboratories, Burlingame, CA) or ProLong Gold Antifade (Thermo Fisher Scientific, Waltham, MA) and mounted on the glass slide for microscopy. For the immunostaining of trans-well membrane, which was used in TEM assay, the staining was done directly in the chamber in 24-well plate. The membrane was fixed with 4% paraformaldehyde and permeabilized. It was blocked with buffer containing purified anti-mouse CD16/32 antibody (Biolegend, San Diego, CA). After antibody staining, the membrane was excised from the insert with a sharp point scalpel and placed on a glass slide. ProLong Gold antifade (Thermo Fisher Scientific, Waltham, MA) and No. 1 coverslip were used for mounting the membrane. For immunostaining of endothelial cells, cells were seeded in an 8-chambered glass slide, which is seeded with 50,000 cells per each chamber. The cells were treated with either PBS or rmCIRP (0.5 µg/mL) for 4 h and then subjected to immunofluorescence staining. Cells were fixed with 4% paraformaldehyde for 15 min at room temperature and washed 3 times with PBS. The fixed cells were permeabilized by 0.1% Triton X-100 (Sigma, St. Louis, MO) for 15 min then blocked with 5% BSA and 100 mM glycine in PBS for 2 h, followed by the incubation of the primary antibody overnight at 4 °C. Primary antibody was mixed in the blocking buffer. The cells were then washed 3 times with PBS and incubated with appropriate secondary antibody for 2 h. After washing the cells 3 times with PBS, the immunofluorescence sample was prepared by adding ProLong Gold Antifade (Thermo Fisher Scientific, Waltham, MA). All slides were observed under the confocal microscope and the staining pattern was quantified. Fiji ImageJ was used for quantification of fluorescence intensity of the immunofluorescence assay.

### Acute lung injury model

C57BL/6 male mice were used in the acute lung injury model. The lung injury was induced by direct intratracheal instilling of lipopolysaccharide (LPS) (Ehrentraut et al. 2019). Briefly describing, mice were anesthetized with 2% isoflurane inhalation prior to intratracheal lipopolysaccharide infusion. A 22G intravenous catheter was used for oral tracheal intubation. 5 µg/g body weight (BW) LPS was delivered into the trachea through the catheter and 50 µL of air was added to ensure that the complete fluid volume was distributed to the lungs. To prevent suffocation, the LPS concentration was adjusted so that the total injection volume was less than 60 µL. Mice treated with LPS were resuscitated subcutaneously with 0.5 mL saline to avoid dehydration caused by surgical stress and then returned to their cages with normal access to food and drinking water. Mice were used for experiment 24 h after LPS instillation.

### Bronchoalveolar lavage and neutrophil isolation

Mice were euthanized with CO_2_ asphyxiation prior to collecting bronchoalveolar lavage (BAL). The throat of a mouse was disinfected with 70% ethanol and the trachea was exposed carefully with scissors and tweezers. The trachea was wrapped around by a suture and then punctured. Venous catheter (22 G) was inserted and fixed with a suture. 0.5 mL of sterile PBS and 0.1 mL air were instilled by a 1 mL syringe through the inserted catheter and then aspirated. The procedure was repeated three times. The cells obtained were then subjected to neutrophil enrichment. Neutrophil enrichment was done with EasySep™ Mouse Neutrophil Enrichment Kit.

### Statistical analysis

Data represented in the figures are expressed as mean ± SEM. A two-tailed Student’s *t*-test was applied for two-group comparison. Significance was considered for p ≤ 0.05 between study groups. Data analyses and the graph preparation were carried out using GraphPad Prism graphing and statistical software (GraphPad Software, San Diego, CA).

## Results

### eCIRP increases neutrophil trogocytosis during TEM

**Fig. 1:**
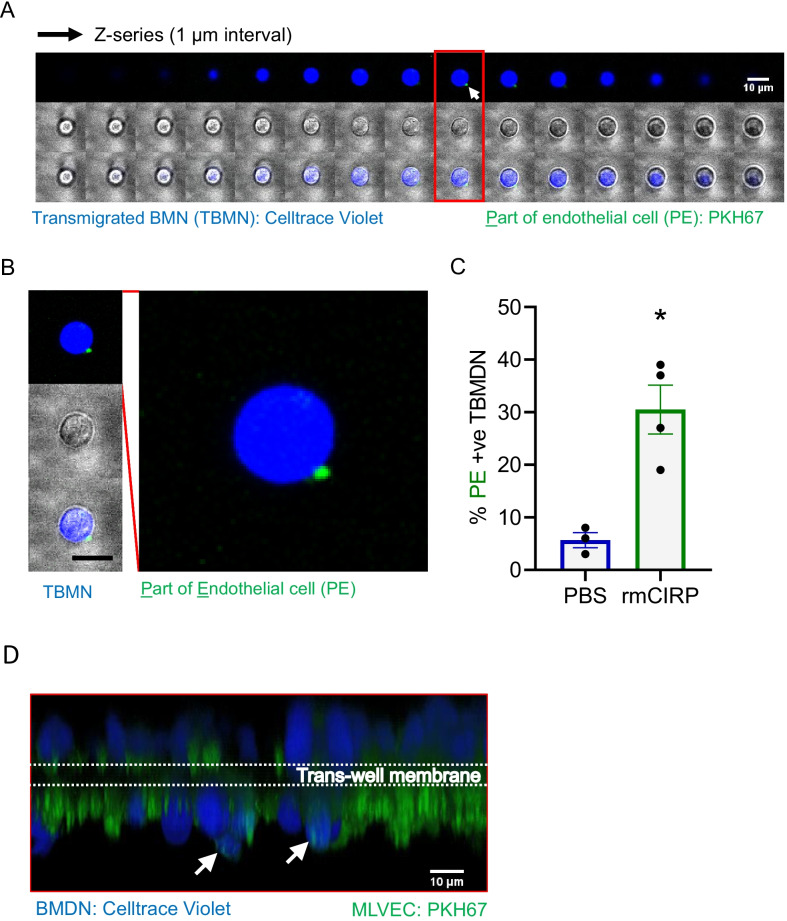
eCIRP increases neutrophil trogocytosis during TEM. Bone marrow neutrophils (BMNs) were stained with CellTrace Violet (blue pseudo color used for illustration) and mouse lung vascular endothelial cells (MLVEC) were stained with green-fluorescent lipophilic membrane staining dye, PKH67, immediately before the assay. **A** The MLVECs were cultured in the transwell insert for 2 days prior to experiment. Then freshly prepared BMNs were added to the insert with either PBS or eCIRP (0.5 µg/mL). The chemoattractant, fMLP (1 nM), was added to the bottom chamber. **A** Montage of z-stack images of the trans-endothelial migrated neutrophils generated with fiji ImageJ. The confocal images were taken by Zeiss LSM880. The arrow indicates the piece of the endothelial cell membrane carried by neutrophils. Scale bar, 10 µm. **B** An enlarged image of a single slice of the z-stack images in **B** (red box) shows green-fluorescent punctum sitting on the blue stained neutrophil. Scale bar, 10 µm. **C** Percent PE positive (+ve) trans-endothelial migrated BMN (TBMN) were quantified by confocal microscopy. **D** Confocal microscopic 3-dimensional reconstruction image of z-stack showing cells migrating through the trans-well membrane in the presence of eCIRP (0.5 µg/mL) at 2 h after treatment. The arrows indicate the endothelial membrane piece carried by the migrated neutrophils. Scale bar, 10 µm. Data represented as mean ± SEM. *p < 0.05, unpaired, two-tailed student’s *t*-test

To investigate the role of eCIRP in neutrophil trogocytosis, neutrophils treated with either PBS or eCIRP were subjected to TEM assay in the presence of the chemoattractant fMLP for 4 h. We then collected the migrated cells from the bottom chamber of the TEM assay. The transmigrated neutrophils were observed on the confocal microscope. The representative serial z-stack images of a transmigrated neutrophil showed a punctate piece of green fluorescence on the surface of the cell (Fig. 1A). The zoomed image of the transmigrated neutrophil showed the green puncta was sitting on the surface of the cells and not co-localized with the cytosolic contents, which is indicated with a blue color in the image (Fig. 1B). The transmitted light image of the cell also showed that the green fluorescence signal sat on the edge of the cell. These data clearly showed that the neutrophils had acquired cellular components from endothelial cells while they transmigrate through the endothelial layer; hence it was clear that trogocytosis had occurred. The cells collected from the bottom chamber of the TEM assay was quantified based on the observation described in Fig. 1A. Treatment with eCIRP showed 30.5% in green fluorescence positive transmigrated neutrophils whereas the cells treated with only PBS showed 5.7%, a significant 5.4-fold increase in neutrophil trogocytosis with eCIRP treatment (Fig. 1C). The data obtained are the average of three (PBS alone) or four (eCIRP treated) biologically independent experiments and each time over 500 neutrophils were observed by the confocal microscope. These data clearly indicate that neutrophils undergo trogocytosis from endothelial cells during paracellular trans-migration and that eCIRP significantly increased trogocytosis in these neutrophils.

**Fig. 2:**
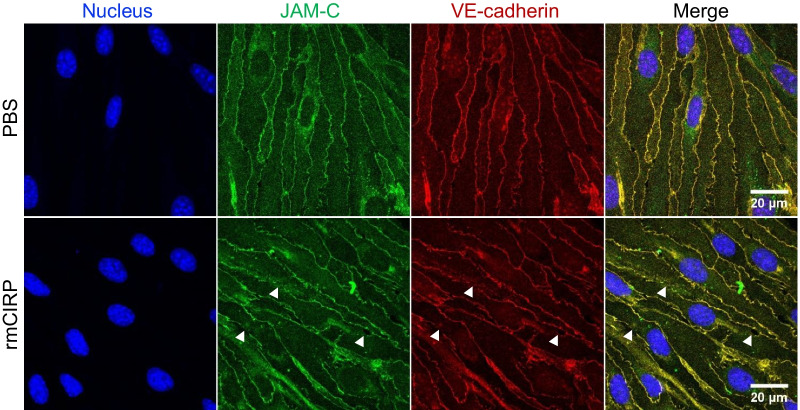
eCIRP decreases cell-to-cell junction of endothelial cells. **A** The endothelial cells cultured on an 8-chambered glass slide were treated with PBS or eCIRP (0.5 µg/mL) for 4 h and were fixed and subjected to immunostaining with antibodies JAM-C (green color) and VE-cadherin (red color). Hoechst33342 was used for nuclear staining and colored in blue. The arrowhead shows junctional disruptions in the endothelial cell layer. Scale bar, 20 µm

To rule out attachment of endothelial vesicles to the neutrophils as opposed to actual membrane transfer/trogocytosis, TEM was conducted with eCIRP treated neutrophils in the presence of fMLP. Confocal live images were taken during neutrophil migration through the transwell endothelial cell layer. The 3-D volume view image reconstructed from the z-stack of confocal microscope images taken after incubation with eCIRP for 2 h showed that the neutrophils took up a small portion of the membrane from the endothelial cells (Fig. 1D). Neutrophils were observed ovoid and bigger than their actual size because of the spill-over of the fluorescence signal. Even though the images were not taken at optimal resolution, we could still observe neutrophils readily migrating through the 3-µm pore size transwell membrane and the green-fluorescent endothelial cell pieces that were accompanied by the neutrophils migrating through the endothelial cell layer. Endothelial cells seeded on top of the transwell membrane usually form a monolayer but some of them do pass through the pores and attached to the bottom of the transwell membrane during the TEM assay. Under the microscope, those cells are seen with higher fluorescence intensity than those on top of the transwell membrane. We also observed that the transwell membrane itself interfered with the fluorescence imaging and significantly decreased the intensity of the light passing through it. Nevertheless, this experiment confirmed that neutrophil trogocytosis does occur during paracellular transendothelial migration in the presence of eCIRP (Fig. 2).

### eCIRP compromises cell-to-cell junction in the endothelial cell layer during neutrophil TEM

**Fig. 3:**
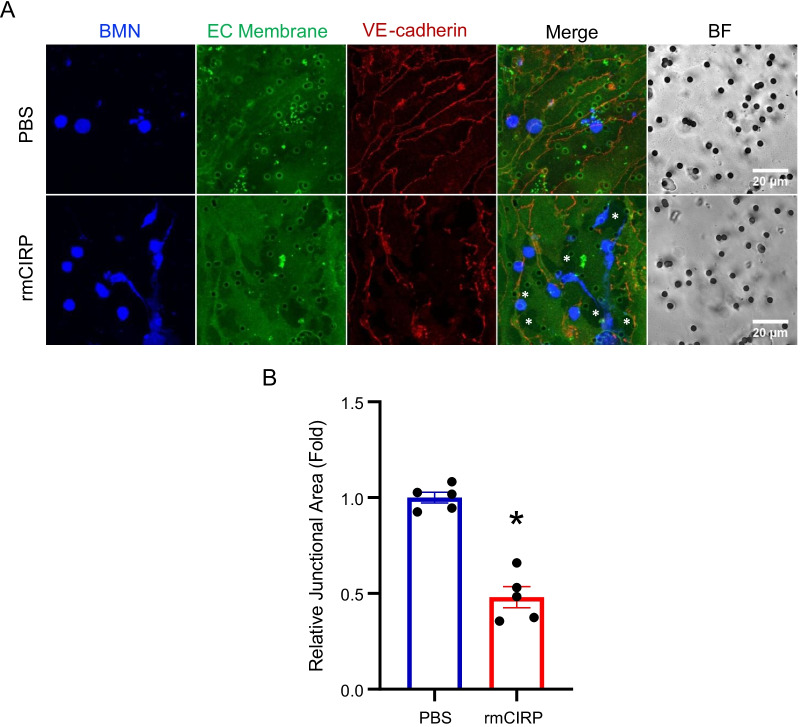
eCIRP compromises cell-to-cell junctional integrity during neutrophil TEM. **A** The TEM assay was performed with either PBS or eCIRP (0.5 µg/mL) for 4 h and transwell membranes were prepared to do the immunofluorescence staining. Attached neutrophils and endothelial cells were directly stained on the transwell membrane with VE-cadherin antibody (red color) and mounted onto a glass slide for microscopy. Neutrophils (BMN) were pre-stained with Celltrace Violet (blue color) prior to the assay. Endothelial cells were stained with fluorescent membrane staining dye, Membrite Green (green color) at the beginning of the TEM assay. Bright field (BF) panel of the images show the pores (black holes; 3 µm in diameter pores) of the transwell membrane. Asterisks (*) in the image for eCIRP treated indicate the regions of transwell membrane, where the endothelial cells are void due to their migration to the bottom of the transwell membrane. Scale bar, 20 µm. **B** The VE-cadherin immunofluorescence was further analyzed by measuring the total area of VE-cadherin in the junctions in 5 different microscopic fields. The data obtained was normalized to the level of PBS treated sample. Data represented as mean ± SEM. *p < 0.05, unpaired, two-tailed student’s *t*-test

To examine the effect of eCIRP in endothelial cell junction we cultured the mouse lung vascular endothelial cells on the glass bottom slides and treated with eCIRP at 0.5 µg/mL for 4 h. The cells were then stained with JAM-C and VE-cadherin antibody and subjected to immunofluorescence microscopy. The cells treated only with PBS were compared to show the effect of eCIRP in the endothelial cells. Both JAM-C and VE-cadherin staining showed lack of staining in junctions indicating eCIRP itself compromised the junctional adhesion. Since neutrophils are known to cause endothelial injuries mediated via soluble mediators which are released from neutrophils (Sarma and Ward 2014), we then checked the effect of the neutrophil and endothelial cell interaction upon treatment of eCIRP during TEM. In the presence of neutrophils, TEM assay was performed with either PBS or eCIRP and the transwell membrane were subjected to the immunofluorescence staining with anti-VE-cadherin antibody. The resulting immunofluorescence images showed significant destruction of cell-to-cell junction in eCIRP treated cells whereas PBS treatment did not aggravate the endothelial cell layer (Fig. 3A). The image analysis and quantification of junctional area of VE-cadherin showed that eCIRP treatment significantly decreased the area to half that of PBS treated cells (Fig. 3B). As a result of the junctional loss in the monolayer of eCIRP treated cells, there were significant increase of clear areas in the microscopic field, which may mimic the injury of blood vessels in vivo.

### eCIRP increases adhesion of neutrophils to endothelial cells

**Fig. 4:**
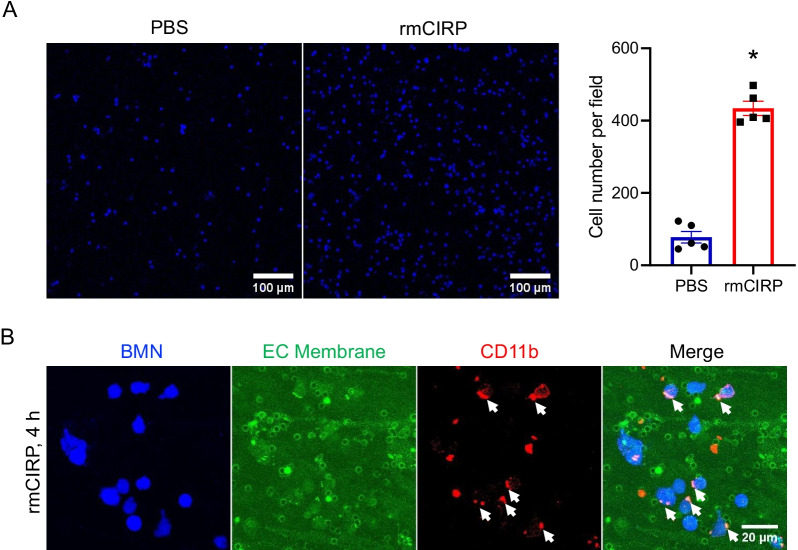
eCIRP increases the adhesion of neutrophils to endothelial cells. **A** Neutrophils on the transwell membrane recovered from the TEM assay were counted by using wide field microscopic images and fiji ImageJ. Scale bar, 100 µm. The cell number of neutrophils per field in PBS or eCIRP treated transwell membrane were quantified. Data represented as mean ± SEM. *p < 0.05, unpaired, two-tailed student’s *t*-test. **B** Representative images of neutrophil adhesion to the endothelial cells showing CD11b on the neutrophils (red color) co-localized with plasma membrane staining signal of the endothelial cells (green color) on the transwell membrane after TEM assay. The arrows in the CD11b and the merge panels indicate polarization of CD11b with the plasma membrane staining on the neutrophils. Scale bar, 20 µm

As we have observed that eCIRP treatment decreases junctional adhesion of endothelial cells and the presence of neutrophils significantly ameliorate cell-to-cell contacts of endothelial cell layer in the TEM assay, we investigated the effect of eCIRP to the adhesion of neutrophils to the endothelial cells. We examined the neutrophils present on the transwell membrane by low magnification objective and could detect a significant increase in neutrophils bound to the endothelial cells on the transwell membrane in response to the treatment of eCIRP. Counting the number of neutrophils in 5 microscopic fields each, we showed that eCIRP treatment increased the number of neutrophils bound to the endothelial cells to be 5.6-fold higher than with the PBS control (Fig. 4A). To examine whether there is a change in integrin localization on the neutrophils bound to endothelial cells on the transwell membrane, we checked the distribution of Mac-1 (CD11b/CD18 heterodimer) on neutrophils. Mac-1 is known to be localized in the mid-section of the neutrophils during the uropod elongation step of extravasation and showed clustering, while high affinity LFA-1 (CD11a/CD18 heterodimer) is localized at the leading edge of extravasating neutrophils (Hyun et al. 2019). The transwell membrane with neutrophils attached to the endothelial cells were stained using CD11b antibody. We observed the clustering of Cd11b on the neutrophils while they were attached to the endothelial cells on the transwell membrane (Fig. 4B). These data suggest the neutrophils treated with eCIRP facilitates clustering of Mac-1, which interacts with membrane molecules on the endothelial cells.

### eCIRP induces neutrophil trogocytosis of JAM-C and VE-cadherin from endothelial cells during TEM

**Fig. 5:**
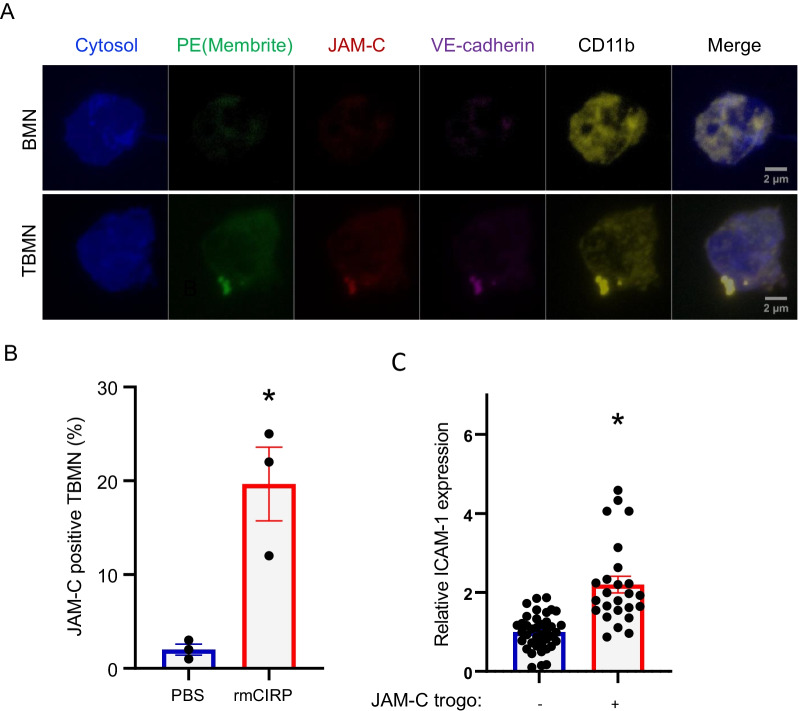
eCIRP induces neutrophils to acquire JAM-C and VE-cadherin from endothelial cells and become pro-inflammatory. **A** The neutrophils trans-migrated through the endothelial cell layer on the transwell membrane (TBMN) and freshly isolated neutrophils (BMN) were further analyzed by immunostaining with antibodies to anti-JAM-C (red color), anti-VE-cadherin (magenta color), and anti-CD11b (yellow color). The neutrophils stained with antibodies were mounted on the glass slide and images were acquired with Zeiss LSM900 Airyscan 2 mode. The neutrophils were pre-stained with Celltrace Violet dye and shown in blue color in the pictures and endothelial cells were stained with PE (membrite) shown in green color prior to the assay. The arrow in the merged panel indicates co-localization of the signals. Scale bar, 2 µm. **B** The trans-migrated neutrophils (TBMN) stained as in the **A** were observed individually under the confocal microscope. JAM-C-positive TBMN treated with eCIRP were compared to the PBS control in the TEM assay. **C** The ICAM-1 expression level was observed by confocal microscopy. The trans-migrated neutrophils were collected and stained with anti-JAM-C antibody and anti-ICAM-1 antibody and the cells were individually imaged. The expression level of ICAM-1 in JAM-C positive neutrophils were compared to the level of ICAM-1 in JAM-C negative neutrophils. Data represented as mean ± SEM. *p < 0.05, unpaired, two-tailed student’s *t*-test

The interaction between Mac-1 of neutrophils and JAM-C at the junctions of blood vessel is a crucial event during the trans-migration of neutrophils (Woodfin et al. 2011). To examine the interaction between Mac-1 and JAM-C during TEM, we collected trans-migrated neutrophils and stained them with CD11b, JAM-C, and VE-cadherin antibodies. The trans-migrated neutrophils carrying the endothelial cell pieces also showed JAM-C, and VE-cadherin staining (Fig. 5A, lower panel). CD11b was localized predominantly to the region of the neutrophil where the endothelial cell pieces were seen, and the staining showed intensely brighter signal than the rest of the area of the neutrophils. Interestingly, this polarization of CD11b on the surface of the neutrophils was not observed in neutrophils which was freshly prepared from mouse bone marrow (Fig. 5A, upper panel). Furthermore, eCIRP treatment significantly increased the JAM-C staining with co-localization of the green staining of the trans-migrated neutrophils. The JAM-C positive trans-migrated neutrophils with PBS treatment was 1.5% and treatment with eCIRP increased this number to 19.7% (Fig. 5B), an average 13-fold increase in JAM-C positive trans-migrated neutrophils with eCIRP treatment.

### Transmigrated neutrophils become pro-inflammatory

We have previously shown that eCIRP increases ICAM-1 expression in neutrophils and ICAM-1 + neutrophils exhibited NETosis. To examine the clear relationship between trogocytosis and procurement of inflammatory phenotype of neutrophils, we analyzed ICAM-1 expression level of JAM-C positive trans-migrated neutrophils. In this experiment, eCIRP treated trans-migrated neutrophils from the TEM assay were collected and stained with anti-ICAM-1 and anti-JAM-C antibodies and individual cells were imaged with the confocal microscope (Additional file 1: Fig. S1). The ICAM-1 expression was significantly elevated in the trans-migrated neutrophils carrying the JAM-C positive endothelial cell pieces on their surface (Fig. 5C). These data suggest that the JAM-C trogocytosis induced by eCIRP makes the neutrophils more pro-inflammatory.

### Neutrophil trogocytosis is observed in injured lung

**Fig. 6:**
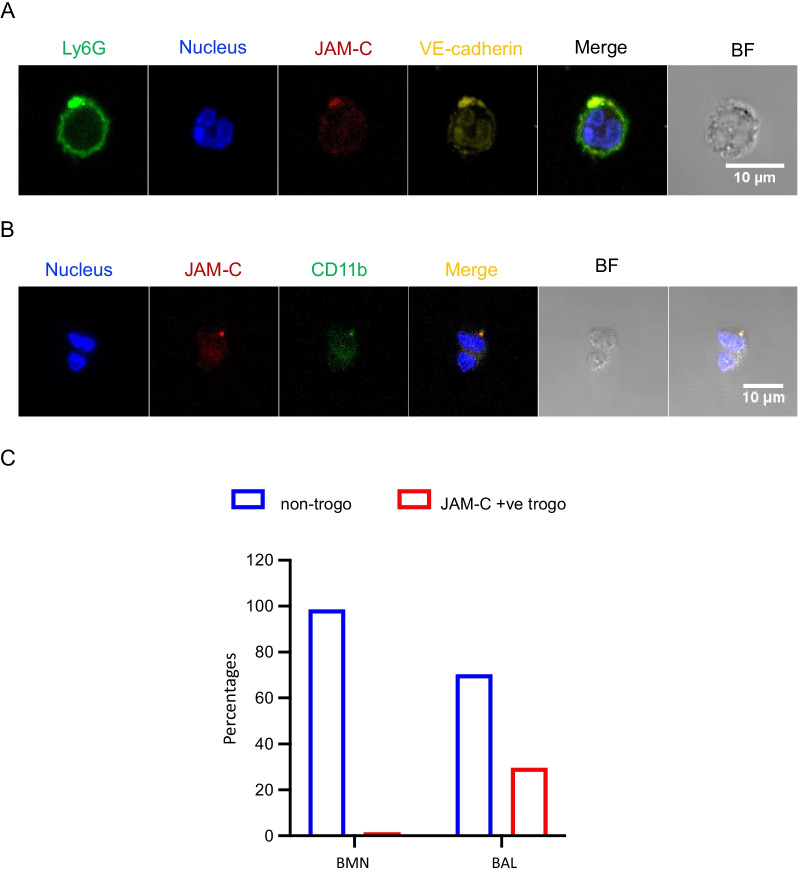
Trogocytosis of neutrophils is observed in injured Lung. The acute lung injury model was used to detect the trogocytosis of neutrophils. The mice were challenged with LPS by direct intra-tracheal instillation. The neutrophils from mouse lungs were collected by bronchoalveolar lavage (BAL). **A** To show the trogocytosis of neutrophils in mouse lung tissue, the purified neutrophils from BAL fluid were subjected to antibody staining with anti-Ly6G, anti-JAM-C, and anti-VE-cadherin antibody. The nucleus of the cells was stained with Hoechst33342. Scale bar, 10 µm. **B** The neutrophils were also subjected to immunofluorescence staining with anti-JAM-C antibody and anti-CD11b antibody to show co-localization of the two markers in the neutrophils. Scale bar, 10 µm. **C** The trogocytosis of purified neutrophils were quantified by observing the cells (over 500 cells each for BMN and BAL neutrophils) with confocal microscopy. The number of trogocytosis was determined by observing JAM-C positive (+ve) puncta on the edge of cells. Data are shown in percentages

To see the presence of trogocytosis of neutrophils in pathophysiological condition, we employed an acute lung injury model by direct instillation of LPS via the trachea. BAL fluid was collected at 24 h after LPS instillation. The trans-migrated cells in BAL fluid were isolated and subjected to immunofluorescence staining. Cells were stained with anti-Ly6G, JAM-C, and VE-cadherin antibodies. Hoechst33342 was used for nuclear staining (Fig. 6A). Ly6G was used as neutrophil positive marker and nucleus staining confirmed the typical nuclear morphology of neutrophils. The presence of JAM-C and VE-cadherin on the edge of the neutrophils was clearly observed in the neutrophils. As in the case where CD11b was being polarized to where the endothelial cell pieces exist, it was noted that the Ly6G showed significant polarization where the JAM-C and VE-cadherin were co-localized on the neutrophils. As shown in Figs. 4B and 5A, CD11b polarization on neutrophils was observed where the endothelial cells form a contact to the neutrophils on the transwell membrane and furthermore, it was still highly polarized where the endothelial cell pieces were located on the trogocytosed trans-migrated neutrophils. Therefore, in the second set of immunofluorescences staining, anti-JAM-C and CD11b antibodies were used to crosscheck what was observed from the in vitro TEM assay (Fig. 6B). In the representative figures of cells isolated from BAL showed co-localization of JAM-C and CD11b as we have observed in the in vitro study. We then quantified the number of JAM-C positive neutrophils in BAL fluid. About 30% of cells were positive for JAM-C staining on the surface of the neutrophils (155 out of 523 total observed cells) whereas the neutrophils isolated from bone marrow showed only 1% of cells were JAM-C positive (7 out of 517 total observed cells (Fig. 6C). These data showed the evidence of neutrophil trogocytosis in vivo during paracellular trans-endothelial migration.

## Discussion

Neutrophil trogocytosis were seen in different modes including cancer cell killing with coated antibody, parasite removal, excess sperm removal, and cellular communication with other immune cells (Uribe-Querol and Rosales 2021). While several studies have shown trogocytosis between neutrophils and immune cells, very little is known about neutrophil trogocytosis with non-immune cells such as endothelial cells. It is well recognized that in response to the signals from the site of infection, circulating neutrophils extravasate to the abluminal space where the infection occurs and subsequently clear the pathogens (Margraf et al. 2021). This TEM of activated neutrophils has been studied extensively (Filippi 2019). However, in this study we showed for the first time that during TEM following inflammation, neutrophils undergo trogocytosis by acquiring a small portion of the endothelial membrane and its contents. We then observed that the trogocytosis of neutrophils was significantly increased by eCIRP stimulation suggesting eCIRP could be a molecular mediator of neutrophil trogocytosis during paracellular transmigration through the blood vessel. In the TEM assay, we observed decreased VE-cadherin expression in eCIRP treatment compared to PBS control indicating significant junctional damage of endothelial cells mediated by activated neutrophils.

We also showed that eCIRP causes direct damage to the endothelial cells as shown by decreased JAM-C and VE-cadherin expression on the endothelial cell junctions. JAM-C is a member of the junctional adhesion molecules family and a critical contributor to neutrophil TEM (Chavakis et al. 2004; Zen et al. 2004). The shedding of JAM-C from the endothelial cells promotes the reverse migration of neutrophils, which can contribute to systemic inflammation (Woodfin et al. 2011; Colom et al. 2015). While junctional damage was observed with eCIRP treatment on the endothelial cells alone, the damage was exacerbated by the presence of the neutrophils. The immunofluorescence analysis of the trans-migrated neutrophils indicated that VE-cadherin and JAM-C co-localized to the site where the endothelial membrane pieces reside on the trans-migrated neutrophils and eCIRP significantly increased JAM-C positive trans-migrated neutrophils. Since JAM-C and VE-cadherin are expressed at distinguished sites between endothelial cell junctions, it can be argued that how these two molecules can be present in the trogocytosed endothelial cell membrane puncta/spec. As shown in Fig. 2, the staining indicate that these two molecules are co-localized at the endothelial cell junctions. In fact, damages to the cell junctions in eCIRP treated group can be seen as decrease in both JAM-C and VE-cadherin staining at the same places in the monolayer. Perhaps, the green endothelial cell membrane spec picked up by the transmigrated neutrophils contains other cell junctional proteins in addition to VE-cadherin and JAM-C. Clearly, additional experiments are needed to confirm this speculation.

Treatment with eCIRP significantly increased neutrophil adhesion to the endothelial cells. As expected, we observed significant clustering of CD11b molecules on the neutrophils while the cells were attached to the endothelial cell layer on the top of the transwell membrane. Prior report showed that eCIRP activates the endothelial cells and increases the expression of ICAM-1 which in turn increases the adhesion of immune cells such as neutrophils and facilitate their extravasation (Yang et al. 2016). Thus, it is conceivable that the increased integrin levels on neutrophils and increased expression of ICAM-1 on the endothelial cells may contribute mutually to the increase in the neutrophil adhesion by eCIRP which in turn contributes to the prolonged polarization of Mac-1 on the neutrophils. Prior studies showed that CD11b^hi^ LDNs were characterized as pro-inflammatory as they express CXCR4, ICAM-1, iNOS and form reactive oxygen species (Takizawa et al. 2021). Previously we have also shown that eCIRP up-regulates the expression of ICAM-1 and that neutrophil NETosis by ICAM-1^+^ neutrophils cause acute lung injury (Ode et al. 2018). We showed that the JAM-C positive trans-migrated neutrophils express more ICAM-1 than JAM-C negative trans-migrated neutrophils. It can be speculated that increased ICAM-1 expression in JAM-C positive trans-migrated neutrophils suggests these neutrophils as pro-inflammatory. Neutrophils collected from the BAL fluid in an acute lung injury model showed co-localization of JAM-C, VE-cadherin and CD11b. Compared to freshly prepared naïve neutrophils, BAL fluid contained increased percentage of JAM-C positive neutrophils. These data confirmed our in vitro findings in an in vivo setting indicating JAM-C positive trogocytosed neutrophils are pro-inflammatory. These data collectively suggest that neutrophils do undergo trogocytosis and eCIRP could promote such response during injury and inflammation.

We have previously shown that intratracheal administration of eCIRP in mice significantly increased NETosis and PAD4 expression in the lungs compared to vehicle injected mice. In vitro culture of bone marrow neutrophils with eCIRP also significantly increased NETosis and PAD4 expression compared to PBS control. Taken together, these data clearly showed in both in vivo as well as in vitro that neutrophils exposed to eCIRP produce NETs (Ode et al. 2019). As shown in Fig. 3, eCIRP treated neutrophils clearly contribute to endothelial cell damage. It would be interesting to see the role of NETs in eCIRP mediated neutrophil trogocytosis. Future studies can address this issue.

eCIRP functions at multiple levels during neutrophil TEM. First, as shown in Fig. 4A, eCIRP increases neutrophil adhesion to the endothelial cell possibly by increasing Mac-1 expression on the neutrophils. We have recently shown that in eCIRP injected mice or in sepsis mice, the frequency and number of Cd11b^hi^ and Cd11b^lo^ low density neutrophils were increased in the blood. Those Cd11b^hi^ low density neutrophils produce more NETs and reactive oxygen species compared to the Cd11b^lo^ counterpart (Takizawa et al. 2021). Therefore, eCIRP could be priming the neutrophils to increase Mac-1 to adhere and migrate more efficiently. Second, noting the massive destruction of junctions in the endothelial layer on the transwell membrane in our study as shown in Fig. 3A, and based on our previous reports that neutrophils release extracellular traps in response to the treatment of eCIRP (Ode et al. 2018, 2019; Jin et al. 2019), we consider that eCIRP activates the neutrophils to release their cellular contents, such as enzymes like elastase. It is possible that eCIRP activated neutrophils release PMN elastase and compromises the endothelial junction facilitating JAM-C and VE-cadherin transfer to the neutrophils. Third, as shown in Fig. 5A TBMN, Mac-1 polarization with JAM-C and VE-cadherin indicates strong interaction of Mac-1 with these junctional adhesion molecules. Thus, eCIRP could potentiate the anchorage between the migrating neutrophils and the endothelial cells facilitating neutrophil trogocytosis. While further mechanistic study is required in future work, we can only speculate that mechanical forces that neutrophils generate during the trans-migration of neutrophils may be the driving force that governs the uptake processes of trogocytosis in neutrophils.

Since fMLP is a potent neutrophil chemoattractant, we used fMLP in the in vitro TEM assay. Using intermediate attractant like MIP-2, KC and TNF-a could rule out the interplay between eCIRP and fMLP. However, since fMLP was present in both PBS and eCIRP treated groups, it is highly unlikely that fMLP alone could be responsible for neutrophil trogocytosis, rather that fMLP facilitated neutrophil transmigration. Using anti-CIRP antibodies one could address the interplay of eCIRP and fMLP in the in vitro TEM assay. While eCIRP has been characterized as a DAMP, it has not been shown yet as a chemoattractant for neutrophil transmigration. We believe that eCIRP’s role is to activate the neutrophils to adhere to the endothelial cells more efficiently as shown in Fig. 4A. It would be interesting to see whether there is any difference if neutrophils or endothelial cells are exposed to eCIRP prior to the TEM assay. Since eCIRP is released in response to infection or inflammation from immune cells such as macrophages, we reasoned those neutrophils in circulation and endothelial cells are being exposed to eCIRP simultaneously. In the in vivo model, LPS administration could cause the release of eCIRP from immune cells in the lungs and activate the neutrophils and in turn, trogocytose from endothelial cells during TEM. It is possible that neutrophil trogocytosis during diapedesis in vivo may not solely be due to eCIRP even though eCIRP plays an important role. However, as shown in Fig. 1A–C, treatment with rmCIRP in vitro showed 30.5% green fluorescent positive transmigrated neutrophils compared to 5.7% of such neutrophils with PBS treatment which clearly indicate that eCIRP has a major role in neutrophil trogocytosis. Furthermore, we acknowledge that in addition to eCIRP, additional DAMPS released from injured cells could contribute to the observed neutrophil trogocytosis in vivo.

It can be questioned that what we observed with eCIRP treatment during neutrophil TEM was true trogocytosis via membrane transfer or membrane uptake and ingestion by the neutrophil. The transmigrated neutrophil in Fig. 1A clearly shows that the green membrane spec was attached on the surface of the neutrophil and was not internalized into the cytoplasm and thus constitutes membrane transfer. In fact, if the spec was to be internalized, the green color would have been co-localized with the blue color of the neutrophilic cytoplasmic contents. Having said that, we have observed several transmigrated neutrophils with the green membrane spec internalized which could account for membrane uptake and ingestion by the neutrophil. We suspect those internalized membrane components are endothelial cell debris/vesicles. Whether those internalized membrane contents have any role in inflammation has not yet been elucidated. Furthermore, the data shown in Fig. 1C are exclusively those transmigrated neutrophils bearing the green membrane spec on the cell surface. The image shown in Fig. 1A is only a representative image and the criteria we have chosen was that a minimum of one green membrane spec should be presented on the surface as to count as trogocytosed neutrophil. As we observed in the confocal images (Fig. 1D) and in the upper part of the TEM membrane (Fig. 3), endothelial cells also pass through the transwell membrane pores during TEM which create big gaps in the eCIRP treated group whereas the endothelial cell monolayer are clearly intact in the PBS treated group. However, if neutrophils were to simply fall through those gaps, it is highly unlikely that they would be carrying a piece of the endothelial membrane with it. As such, our data do not support the “fall-through” theory.

There are several limitations for the current study. First, the imaging was done in a static fixed state without flow which could bring in confounding variables leading to over interpretation of the results obtained. To rule out this notion, TEM assay was conducted, and confocal live images were collected, and the Z-stack images were reconstructed. This experiment confirmed that the green endothelial cell membrane puncta/spec was indeed picked up by the neutrophils from endothelial cells during TEM. A second drawback is that the observation is limited to high resolution confocal microscopy. Although intravital microscopy could provide higher resolution microscopy on a single cell level, the observed staining of JAM-C and VE-cadherin, two endothelial junctional proteins, in the neutrophils isolated from the in vivo model of infection/inflammation clearly suggest that transmigrated neutrophils in the BAL did undergo trogocytosis. Third, the in vivo lung model uses LPS rather than eCIRP which could indicate that transmigration alone in response to infection, injury or inflammation might lead to trogocytosis events of endothelial membrane components under certain circumstances. We have recently shown that mice deficient in CIRP are protected from sepsis and acute lung injury and that healthy mice injected with rmCIRP develop lung injury (Khan et al. 2017). Although previous work by us have shown the effect of eCIRP on lung injury and inflammation, we have chosen the intratracheal LPS instillation model to induce acute lung injury more accurately as described in the literature (Li et al. 2021). Since it is well regarded that neutrophil transmigration occurs in the lungs during injury and inflammation, we chose to use the lung model for the study. Intratracheal instillation of eCIRP can delineate the precise role of eCIRP in transmigration in vivo. Future studies can address such a possibility. Another limitation is that if the neutrophil performs diapedesis in a transcellular fashion as opposed to paracellular manner, neutrophil trogocytosis of junctional proteins such as VE-cadherin and JAM-C could not occur. We fully recognize this as a major limitation of our study. Additional studies are required to determine the role of eCIRP, if any, in neutrophil trogocytosis during diapedesis via transcellular fashion. We have not addressed whether eCIRP mediated neutrophil trogocytosis is endothelial cell specific. Since neither VE-cadherin nor JAM-C are expressed by neutrophils, the presence of these proteins in the green puncta/spec strongly suggests neutrophil trogocytosis from endothelial cells. Using artificial cell layer such as HELA or Cos7 could certainly determine whether trogocytosis is specific to endothelial cells. Additional evidence is necessary for such a conclusion.

## Conclusion

In summary, we demonstrated for the first time that neutrophils trogocytose from endothelial cells during paracellular trans-endothelial migration and that eCIRP promotes such occurrence in response to inflammation and acute lung injury. We also identified JAM-C from the endothelial cells as a potential contributor of eCIRP induced trogocytosis and that JAM-C positive trogocytosed neutrophils are pro-inflammatory. However, if the JAM-C positive trogocytosed neutrophil which turned pro-inflammatory could in fact reverse migrate and contributes to systemic inflammation during infection/inflammation has yet to be elucidated.

## Supplementary Information

**Additional file 1: Fig S1.** TEM assay was performed with rmCIRP treatment and transmigrated neutrophils were stained with [A] JAM-C (green color) and [B] ICAM-1 (red color) antibodies. JAM-C is expressed by the trogocytosed neutrophil whereas ICAM-1 was seen in majority of the transmigrated neutrophils.

## Data Availability

All data generated or analyzed during this study are included in this article.

## Abbreviations

BAL: Bronchoalveolar lavage
BMN: Bone marrow neutrophil
CD4: Cluster differentiation 4
CXCR4: C-X-C Motif Chemokine Receptor 4
DAMP: Damage associated molecular pattern
eCIRP: Extracellular cold inducible RNA binding protein
fMLP: N-formyl-Met-Leu-Phe
ICAM-1: Intercellular adhesion molecule-1
iNOS: Inducible nitric oxide synthase
JAM-C: Junctional adhesion molecule-C
LPS: Lipopolysaccharides
MHC-II: Major histocompatibility antigen-II
MLVEC: Mouse primary lung micro-vascular endothelial cells
NETs: Neutrophil extracellular traps
PBS: Phosphate buffered saline
RNA: Ribonucleic acid
rmCIRP: Recombinant murine cold inducible RNA binding protein
TBMN: Trogocytosed bone marrow neutrophil
TEM: Trans-endothelial migration
TCR: T cell receptor
