# Supplementary material for: Neutrophil trogocytosis during their trans-endothelial migration: role of extracellular CIRP
Source: Mol Med. 2022 Aug 8;28:91. doi: 10.1186/s10020-022-00515-3 (PMC9358840; doi:10.1186/s10020-022-00515-3)
Supplement: Supplementary file 1 — Additional file 1: Fig S1. TEM assay was performed with rmCIRP treatment and transmigrated neutrophils were stained with [A] JAM-C (green color) and [B] ICAM-1 (red color) antibodies. JAM-C is expressed by the trogocytosed neutrophil whereas ICAM-1 was seen in majority of the transmigrated neutrophils. [file 10020_2022_515_MOESM1_ESM.pptx]

## Slide 1
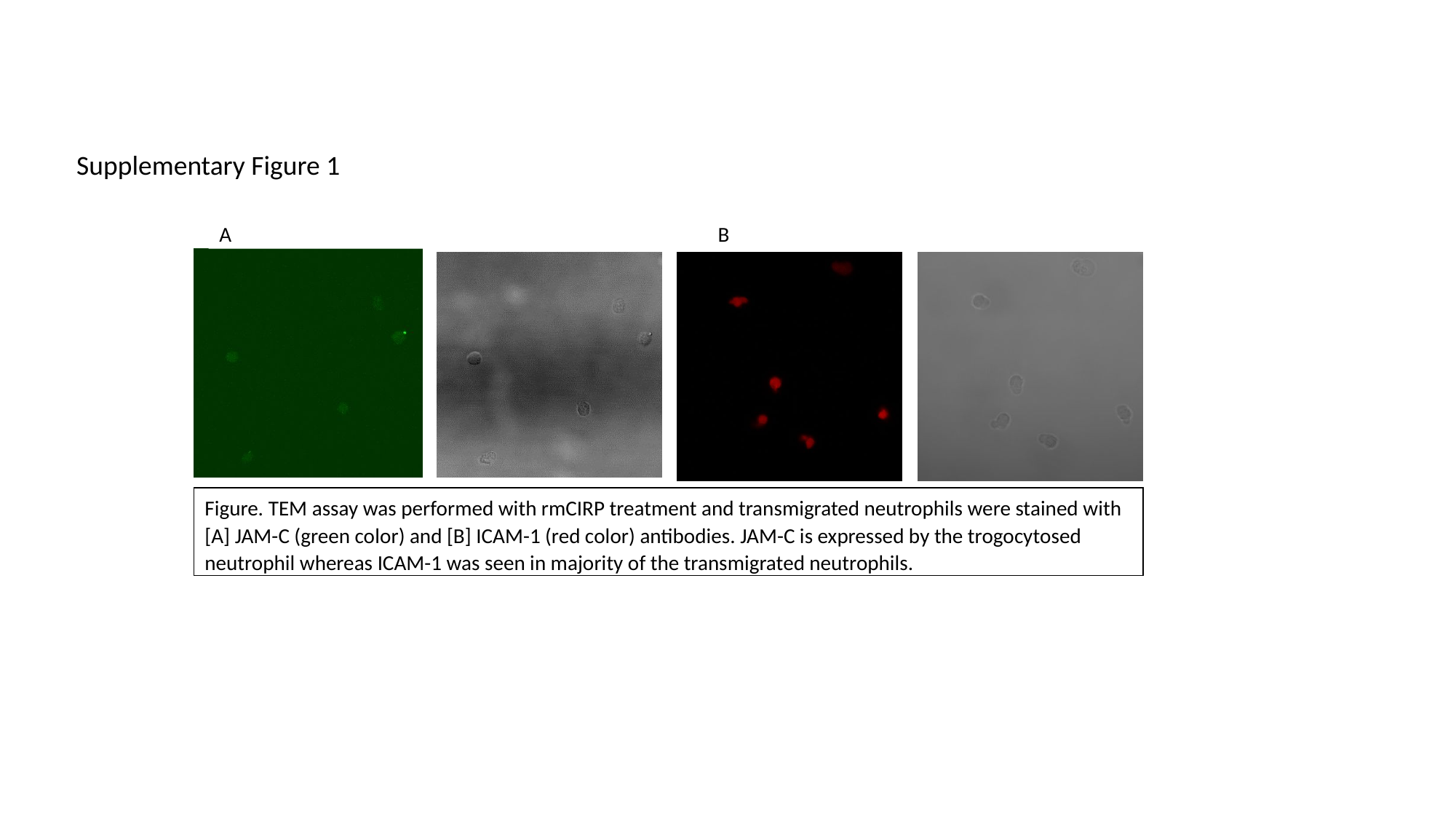

Supplementary Figure 1
A B
Figure. TEM assay was performed with rmCIRP treatment and transmigrated neutrophils were stained with [A] JAM-C (green color) and [B] ICAM-1 (red color) antibodies. JAM-C is expressed by the trogocytosed neutrophil whereas ICAM-1 was seen in majority of the transmigrated neutrophils.
